# Prevention of Infectious Diseases

**Published:** 1896-09

**Authors:** 


### PREVENTION OF INFECTIOUS DISEASES.

Dr. J. M. G. Walker, of Waukegan, Ill., thus concludes a paper in the *Journal of the Amcrican Medical Association* :

1. Systematic cleanliness should be practiced, (a) by thorough disinfection of the patient, the sick room, all instruments, vessels, and other apparatus or clothing in use; (b) by allowing no unclean or infected fabric or vessel to be taken from the room until rendered aseptic.

2. Prevent individual contact (a) by isolation, segregation or quarantine; (b) by prohibiting communication between the infected and the uninfected, exeept under strict surveillance of the physician, or in accordance with his explicit directions.

3. Provide thorough and systematic ventilation of cellars and basements as well as all other rooms in houses used for dwellings, and give free access of air under floors of houses which have no cellars or basements, and supply complete drainage.

4. Fortify the system against pathogenic bacteria (a) by abundant and suitable nourishment; (b) by insisting upon the observance of the laws of hygiene relating to clothing, eating, exercise and bathing; (c) by the administration of certain drugs, among which belladonna, iron and pilocarpine are prominent; (d) by the conservative use of vaccination, inoculation, the serums or antitoxins and perhaps protonuclein.

5. The State or city should see that the poor have work, food, clothing, good shelter, public baths and fresh air.

6. Health inspection officers should keep their wards in good sanitary condition, and see that the water and food supplies are uncontaminated.

